# Swimming smarter, not harder: fishes exploit habitat heterogeneity to increase locomotor performance

**DOI:** 10.1242/jeb.247918

**Published:** 2025-02-20

**Authors:** Valentina Di Santo, Elsa Goerig

**Affiliations:** ^1^ Scripps Institution of Oceanography, University of California, San Diego, La Jolla, CA 92093, USA; ^2^ Museum of Comparative Zoology, Harvard University, Cambridge, MA 02138, USA

**Keywords:** U-shaped curve, Aquatic locomotion, Energetics, Locomotion, Swimming, Hydrodynamics, Habitat restoration

## Abstract

Quantifying the intricate relationship between locomotion, energy expenditure and environmental heterogeneity is pivotal for elucidating the ecological and evolutionary changes in locomotor performance in fishes. This Commentary synthesizes existing research to offer a perspective on how fishes actively exploit complex environments to enhance their locomotor efficiency. Contrary to conventional portrayals of fishes as passive responders to environmental stressors, empirical evidence supports the idea that fishes employ sophisticated strategies to navigate diverse hydrodynamic landscapes. Here, we show clever ways fishes bend the rules of a non-linear speed–energetics curve to save energy below and above optimal cruising speeds. The findings from these behavioral adjustments hold broader implications for understanding fish performance under dynamic environments and conserving fish populations.

## Teaser

**Summary:** Fishes actively exploit complex environments to enhance locomotor efficiency, revealing sophisticated strategies to navigate diverse hydrodynamic landscapes and highlighting the need for habitat complexity in conservation.

## Introduction

The evolutionary success of fishes has been profoundly influenced by their locomotor abilities (Blake, 2004; Hunter, 1998). Swimming is a nearly ubiquitous activity in the life of fishes – encompassing migration, mating, foraging, predator evasion, habitat exploration and hovering – suggesting that a significant portion of the daily energy budget is devoted to locomotion. The intricate dynamics of this energy allocation are complicated by the fact that nearly every physiological process in fishes, and therefore, also swimming physiology and biomechanics, is affected by abiotic factors (Fry, 1958; Neubauer and Andersen, 2019; Schulte et al., 2011). The environmental conditions experienced by fishes are subject to both short-term variations and long-term shifts, the latter being exemplified by the ongoing changes attributed to a changing climate (Somero, 2010; Fields et al., 1993). Moreover, anthropogenic activities are altering the chemical properties of not only aquatic environments but also physical structures, reducing complex 3D landscapes into flat terrains (Di Santo et al., 2020). In fact, fishes can adjust their locomotor performance in response to environmental changes and are influenced by the heterogeneous properties of the fluid dynamics they encounter (Cote and Webb, 2015; Webb et al., 2010; Taguchi and Liao, 2011; Marcoux and Korsmeyer, 2019). Fishes demonstrate a capacity to use complexity and variation in their habitat as a strategy to harness specific conditions to improve their physiological performance (Di Santo and Bennett, 2011a; Farrell et al., 2003; Jørgensen et al., 2016; Kitagawa et al., 2004; Wegner et al., 2015). For instance, fishes can select different temperatures to slow down processes such as digestion or to increase the rate of others such as muscle contraction (Fry, 1958). Additionally, they can exploit complex hydrodynamics and three-dimensional landscapes to reduce energy expenditure while traversing challenging areas, for instance, in rivers and on reefs (Liao, 2007; Johansen et al., 2008). So, although fishes have been long perceived as mere pawns of their environment – a viewpoint encapsulated by the phrase ‘die Spielballe der Umgebung’, or the puppets of the environment (Krehl and Soetbeer, 1899) – we present studies that suggest a far more complex interaction between fishes and their physical habitat. Through a combination of evolutionary adaptations and behavioral strategies, fishes have developed the ability to not only survive, but also thrive in a variety of flow conditions (Langerhans and Reznick, 2010; Binning et al., 2015; Roche, 2021). These adaptations highlight the evolutionary success of fishes and underscore the significance of locomotor abilities as a central factor in this success (Di Santo et al., 2021; Lauder, 2015).

### Non-linear speed–energetics curve for underwater locomotion

**Fig. 1.**
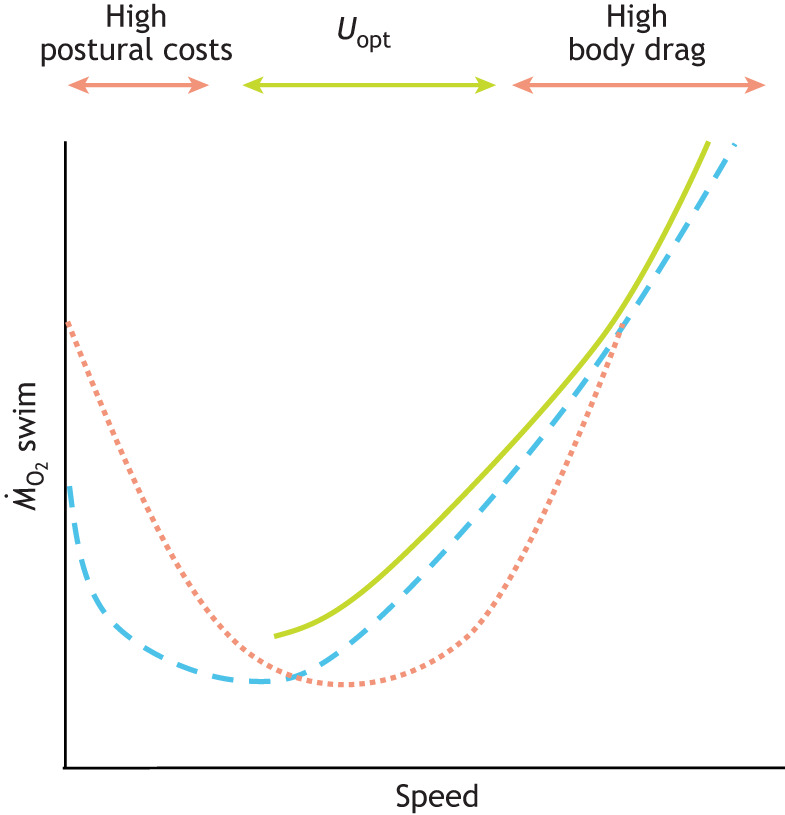
**Different speed–energetics curves.** The relationship between swimming speed and metabolism in fishes can exhibit various patterns: a U-shaped curve (dotted orange line) in negatively buoyant species, a J-shaped curve (dashed blue line), or a linear relationship (continuous green line) in near-neutrally buoyant species. Hydrodynamic theory predicts a non-linear relationship, with metabolic costs increasing at both low and high speeds. At low speeds, postural instability and induced drag elevate oxygen consumption (*Ṁ*_O_2__), whereas at high speeds, body drag becomes the primary contributor to increased *Ṁ*_O_2__. An intermediate range of speeds (optimal speed, *U*_opt_) is expected to be the most energetically efficient. However, most empirical studies show a linear increase in metabolic rates with swimming speeds.

Understanding how fishes adjust their locomotor behavior to conserve energy requires examining the mechanics and energetics of swimming across various speeds. The energetics of flight in negatively buoyant animals, such as birds and insects, typically follow a U-shaped curve, with minimal metabolic rates at intermediate speeds but increased costs at both lower and higher speeds owing to biomechanical and physiological constraints (Ellington, 1985; Tucker and Parrott, 1970). Similarly, hydrodynamic theory suggests that fish swimming should exhibit a non-linear speed–metabolism relationship, with energy costs minimized at intermediate cruising speeds (optimal speeds, *U*_opt_; Tudorache et al., 2011, Dickson et al., 2012) and elevated at both low and high velocities (Webb, 1994; Di Santo et al., 2017; Fig. 1). Despite the buoyancy provided by water, most fishes are inherently unstable because their center of mass is separated from their center of buoyancy, which causes postural challenges, particularly at lower speeds (Webb, 2005; Webb and Weihs, 2015). Stabilization, achieved through fin and body movements, adds to the metabolic costs of swimming, with postural control estimated to account for approximately 10% of total energy expenditure during steady locomotion (Webb and Weihs, 2015). The evidence supporting non-linear speed–metabolism curves has been observed particularly in species that struggle with maintaining stability at low speeds. For instance, Sepulveda et al. (2003) demonstrated that Pacific bonito (*Sarda chiliensis*) exhibit higher metabolic rates at low speeds because of their struggles to maintain steady swimming, and ‘erratic behavior’. Di Santo et al. (2017) observed a non-linear metabolic response in the negatively buoyant clearnose skate (*Raja eglanteria*) and the near-neutrally buoyant rainbow trout (*Oncorhynchus mykiss*) during steady swimming, supporting the hypothesis of U- and J-shaped speed–metabolic curves.

However, to date, the majority of empirical studies show a linear or exponential increase in metabolic rates with speed, rather than the hypothetical U-shaped curve (for example, Brett, 1972; Fulton et al., 2013; Korsmeyer el al., 2002; Ohlberger et al., 2005; Fig. 1). The frequent observation of linear relationships in swimming performance may stem from experimental conditions (Rees et al., 2024). In flow tank set-ups that prioritize high fish mass:water volume ratios, fishes often swim near walls or while touching the bottom of the tank, reducing postural instability and energy expenditure at low speeds, possibly masking non-linear trends (Fish, 2010; Blevins and Lauder, 2013). Larger tanks offer more accurate kinematics data on natural swimming but, at the same time, introduce signal-to-noise issues in metabolic measurements, especially at lower speeds, requiring longer sampling periods, and a slope of the oxygen decline with *r*^2^>0.9 should be attained to ensure precision (Svendsen et al., 2016; Wardle et al., 1996). Small tanks are linked to faster fatigue at higher speeds, whereas at lower speeds, spontaneous activity and ‘restlessness’ can lead to metabolic spikes, complicating data interpretation (Brett, 1964; Rummer et al., 2016; Kern et al., 2017). Faster flows may promote stronger rheotaxis, making fishes orient towards the flow and swim more steadily, thus reducing erratic behavior. Acclimation times prior to the experimental trials may also vary widely in experiments, generally from 12 h (Di Santo and Bennett, 2011b), 2–8 h (Di Santo and Kenaley, 2016; Rummer et al., 2016) to a few minutes (Ashraf et al., 2024), possibly affecting oxygen consumption rates. These factors, along with variation in experimental set-ups, may explain differences in the speed–metabolism relationships observed across studies.

That said, fishes can employ various strategies to conserve energy during locomotion. In the wild, fishes navigate complex environments where factors such as flow direction, velocity and the presence of other organisms can alter fluid dynamics and impact swimming efficiency. This provides challenges as well as opportunities to exploit the physical environment to save energy during locomotion. In this Commentary, we present ways in which fishes can reduce the costs of locomotion at low and high speeds and provide reasoning on what we can learn from these examples to restore complexity in nature as one of the strategies to conserve species.

## Swimming smarter, not harder

Fishes exemplify the principle of ‘swimming smarter, not harder’ by harnessing vortices from other organisms, using the interaction between the flow and 3D structures in their environment, and modulating the movement of body and fins to conserve energy, thereby optimizing their energy expenditure during locomotion.

### Saving energy at low speeds: rolling, surfing and walking

**Fig. 2.**
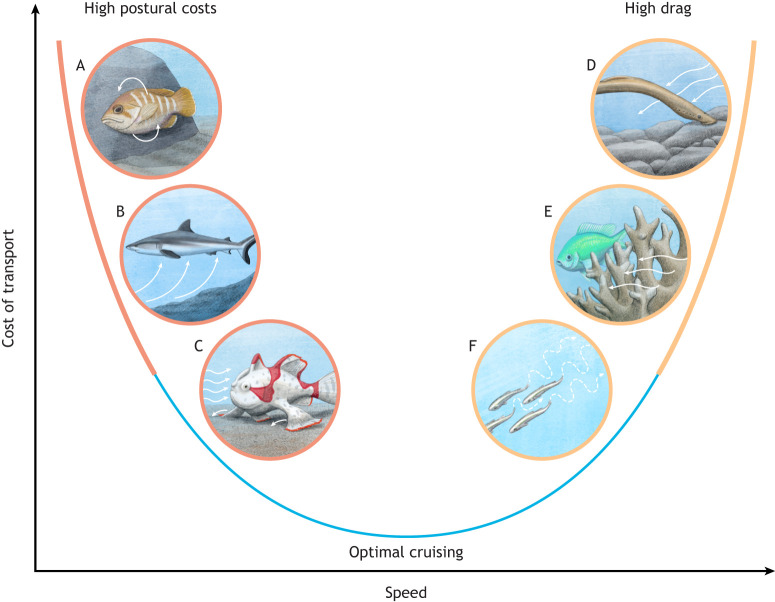
**Swimming smarter, not harder.** Fishes experience increasing high postural costs when swimming below optimal cruising speeds, and high drag when swimming at high speeds. Here, we provide a few representative examples of solutions taken by different fishes to reduce the cost of transport while swimming at low or high speeds. At low speeds, fish might overcome high postural instability by (A) rolling (Argentine sea bass; Ciancio et al., 2016), (B) hovering in areas of updraft (grey reef shark; Papastamatiou et al., 2021) or (C) walking using their pectoral and/or pelvic fins (frogfish; Dickson and Pierce, 2018) instead of swimming to save energy. At high speeds, fishes experience high body drag, so they may (D) attach to structures (lamprey; Beamish, 1973), (E) shelter behind coral to experience lower velocity flow (damselfish; Johansen et al., 2008) or (F) swim in a school formation to take advantage of vortices and reduce the effort of movement (forage fish; Weihs, 1973). Illustrations by Alex Boersma.

At low speeds, most fishes face challenges in maintaining a dorso-ventrally upright posture owing to inherent instability (Webb and Weihs, 2015). To counteract the rotational forces resulting from the physical separation of the centers of mass and buoyancy, fishes must continuously move their fins and adjust their posture. Therefore, fishes may opt to forgo an upright posture and instead roll, utilize updraft zones for surfing, or may even alter their locomotion strategy, switching from swimming to walking. The Argentine sea bass (*Acanthistius patachonicus*), with its laterally compressed body, is affected by rolling along the anterior–posterior axis. This species may minimize energetic costs by relaxing posture control mechanisms during periods of rest (Ciancio et al., 2016). Notably, when inactive, especially within cave refuges, the Argentine sea bass often adopts extreme roll angles exceeding 80 deg (Fig. 2A). This behavior contrasts sharply with other fish species such as the Atlantic cod (*Gadus morhua*) and the great sculpin (*Myoxocephalus polyacanthocephalus*), which neither exhibit such pronounced body compression nor utilize cave refuges for resting. These species typically maintain a more upright posture even during rest. By assuming such extreme roll angles, the Argentine sea bass likely achieves a reduction in the energy required to maintain an upright position during rest periods, thus potentially lowering their overall metabolic rate when compared with hovering (Ciancio et al., 2016).

In a study of energy expenditure strategies among a large negatively buoyant fish (grey reef sharks, *Carcharhinus amblyrhynchos*) within the Fakarava Atoll (French Polynesia), researchers combined direct observations, acoustic telemetry and biologging to elucidate the relationship between shark aggregations, hovering and environmental hydraulics (Papastamatiou et al., 2021). The focal point of that study was to ascertain the extent to which sharks may exploit natural updrafts created by tidal currents against submarine slopes to optimize their energy usage, and effectively surf the slope, at low ground speeds (Fig. 2B). During incoming tides, grey reef sharks exhibit a shuttling behavior, where individuals cyclically move to the front of aggregations to be passively transported to the rear by the current, maintaining their position within these energetically favorable updraft zones without active swimming (Papastamatiou et al., 2021). This behavior diminishes during outgoing tides when the updrafts are no longer found. The biomechanical analysis performed indicated that residing within these updraft zones could lead to a reduction in routine metabolic rates by approximately 10–15% (Papastamatiou et al., 2021). The physiological and ecological implications of these findings suggest that the ability to minimize energy expenditure through clever use of the energetic landscape is a critical adaptive trait in the evolutionary ecology of fishes.

Several groups of fishes – including sharks, skates, frogfishes, lungfishes, bichirs and others – use a combination of pectoral and/or pelvic fins to walk slowly on the substratum. These fishes navigate through complex habitats, increasing benthic foraging efficiency and maintaining postural stability on the ground even when facing strong currents. Epaulette sharks (*Hemiscyllium* spp.), for example, utilize their pectoral and pelvic fins to walk across tidal pools in Australia and New Guinea, which is particularly useful during low tides, when swimming may be ineffective (Porter et al., 2022; Goto et al., 1999). Similarly, frogfishes, belonging to the family Antennariidae, walk on the substratum using their modified pelvic and pectoral fins to move slowly across the seabed, aligning with their ambush predation strategy (Fig. 2C). Skates walk, or ‘punt’, on the substratum using their modified pelvic fins, which is advantageous when the fish wants to explore the environment slowly and efficiently (Ebert and Bizzarro, 2007; Di Santo, 2019). In fact, even though swimming metabolic rates are relatively low in skates, batoid swimming is fairly inefficient when the fish needs to cover long distances (Di Santo and Kenaley, 2016). As body posture control may make up approximately 10% of the energetic expenditure during swimming (Webb and Weihs, 2015), fishes may walk as a way to cut down on the energy costs of stabilizing their bodies at low speeds while still being able to explore and interact with the substratum. However, benthic locomotion is also advantageous in high-flow and turbulent conditions, because it enables fishes to adhere to the substrate for station holding and minimize pressure waves that could reveal their presence to predators (Fox et al., 2018; Lucifora and Vassallo, 2002; Bilecenoglu and Ekstrom, 2013).

### Saving energy at high speeds: hitchhiking, drafting and gliding

At high speeds, fishes need to overcome a significant increase in drag. To conserve energy at high speeds, fishes may opt to ‘cheat’ and attach to other moving animals or rocks, and swim behind structures and other fish to experience lower speeds and save energy.

Remoras are renowned for their unique commensal relationship with larger and faster marine organisms, including sharks, turtles and cetaceans. This association allows remoras to conserve energy that would otherwise be expended on swimming and to capitalize on feeding opportunities created by their hosts' activities (Brunnschweiler and Barnett, 2013). This relationship primarily involves the remora attaching to the host with the aid of a modified dorsal fin that functions as a powerful suction disk, enabling them to hitchhike through the water (Cohen et al., 2020; Flammang and Kenaley, 2017). The modified dorsal fin of the remoras has a series of lamellae that can create a substantial suction force when pressed against a surface, allowing the fish to maintain a firm hold on its hosts even at high speeds (Fertl and Landry, 1999; Flammang and Kenaley, 2017). This attachment mechanism not only facilitates energy conservation but also reduces the hydrodynamic drag experienced by the remora, further enhancing its energy efficiency (Dewar et al., 2011). Energy conservation is a critical benefit of this hitchhiking behavior (Fish, 2010).

Similarly, lampreys attach to structures in the environment to save energy and rest between swimming bursts (Fig. 2D). Lampreys display a remarkable migratory behavior that is important for their survival and reproduction. These fishes, known for their unique parasitic lifestyle during adulthood, undertake extensive freshwater migrations to spawn (Moser et al., 2015). Throughout these migrations, they cease feeding, making energy conservation crucial. A distinctive feature observed in lampreys during these journeys is their use of an oral suction disk to attach to stable substrates such as stones. This highly specialized anatomical adaptation allows them to adhere to smooth surfaces, particularly during upstream migrations, and maintain their position in the river without expending energy to swim against the current. This behavior reduces their metabolic rate (Beamish, 1973), allowing them to rest and conserve energy for the reproductive phase of their life cycle (Quintella et al., 2009).

Lampreys are not the only fishes that can attach to the substratum to hold position in high-flow environments. Waterfall-climbing gobies and northern clingfish exhibit remarkable adaptations for adhering to and climbing on challenging surfaces. Gobies utilize a specialized pelvic sucker, formed by fused pelvic fins, to generate the necessary suction force to support their body weight against both gravity and water drag. This allows them to inch up rock surfaces using alternating movements of their mouth and pelvic sucker (Maie, 2022). The pelvic muscles generate negative pressure within the sucker, creating robust adhesion, which is critical during the transition from water to air where buoyancy is lost, necessitating greater force for adhesion (Schlosser, 1991; Maie, 2022). Similarly, northern clingfish have evolved an adhesive disk capable of adhering to a wide range of surface roughness, from fine sandpaper to highly irregular surfaces (Wainwright et al., 2013). The clingfish's disk, comprising elements of the pectoral and pelvic girdles, features a rough surface with small papillae that enhance its adhesive capabilities. These fish can generate suction forces corresponding to pressures 0.2–0.5 atm below ambient, which are 80–230 times their body weight (Wainwright et al., 2013). This impressive adhesion is facilitated by hierarchically structured microvilli around the disk's edges, resembling the setae on the feet of geckoes and spiders. This microstructure allows clingfish to outperform manufactured suction cups, adhering effectively even to fouled and slippery surfaces (Ditsche and Summers, 2019).

Salmonids undertake migrations that are essential for spawning and growth. During these migrations, particularly upstream, conserving energy is a fundamental adaptive strategy (Liao et al., 2012). One key behavior observed among these migrating trout is their tendency to swim close to or directly behind large stones in rivers. This behavior allows the fish to leverage the hydrodynamic conditions created by these natural roughness elements on the stream bed (Liao, 2006). The hydrodynamics of river flows involve thickening the boundary layer and creating velocity gradients that result in areas of reduced velocity, known as wake zones, immediately downstream of obstacles (Liao, 2006; Goerig et al., 2016; Vogel, 2020). These zones are characterized by von Kármán street vortices, a pattern of swirling vortices generated by the flow of fluid around the obstacles (Liao et al., 2003a,b). Salmonids strategically utilize these wake zones to rest and conserve energy during their upstream migrations. By positioning themselves within these zones, they decrease the physical effort required to maintain their position against the current or even propel themselves forward by ‘surfing’ the vortices, thus reducing their metabolic expenditure (Liao, 2007). This energy conservation strategy is crucial as it allows more resources to be directed towards growth and reproduction, which are especially energy-demanding processes (Standen, 2001; Hinch and Bratty, 2000; Goerig et al., 2017; Liao, 2007). Furthermore, juvenile Atlantic salmon (*Salmo salar*) utilize the riverbed's substrate, such as boulders, to reduce energy expenditure when feeding on drifting invertebrates. In areas with faster currents, such as riffles, the drifting rate of potential food is higher, but it is energetically costly for small salmon to maintain their position in fast flows. By using the boundary layer just above large stones and anchoring themselves with their large pectoral fins, they can maintain position with minimal energy use, darting forward occasionally to catch prey (Wánkowski and Thorpe, 1979). These foraging behaviors are also influenced by social hierarchies, with dominant fish defending optimal locations for substrate cover and food availability, which impacts the growth rates and spatial distribution of the individuals within their populations (Harwood et al., 2003). However, the energetic benefits of this sheltering behavior will depend on the flow velocity gradient and the spatial distribution of the drifting invertebrates. A combination of sheltering and swimming in the freestream while foraging may produce the largest benefit. The strategic use of natural river structures by salmonids illustrates not only an important aspect of river ecology – how the physical heterogeneity of habitats influences behavioral and survival strategies of aquatic organisms – but also how these strategies can be affected by environmental changes. Human alterations to river landscapes, such as damming and river straightening, can disrupt natural flowfields and the availability of hydraulic shelters, potentially affecting the migration efficiency and survival rates of salmonid populations (Young, 1994; Johansen et al., 2020).

Coral reefs provide a complex structural habitat that helps shelter fishes from high-energy environments (Fulton and Bellwood, 2011; Johansen et al., 2007). Fishes may use reef structures to conserve energy (Jones and McCormick, 2004; Johansen et al., 2008; Cotel and Webb, 2012; Schakmann and Korsmeyer, 2023). In fact, the varying current speeds and directions characteristic of coral reef environments significantly influence the energy expenditure of resident organisms (Binning et al., 2014; Fulton and Bellwood, 2011). Species such as clownfish, parrotfish and damselfish exploit the topography of coral reefs to minimize energy costs (Fig. 2E). These reef structures, with their crevices, overhangs and caves, provide sheltered areas where water flow is considerably reduced, allowing fishes to save energy while maintaining position and resisting currents (Fulton and Bellwood, 2011). This behavior is particularly evident during periods of high water flow, such as tidal changes or during storm events, where fishes that effectively use these structures exhibit a marked reduction in metabolic rates (Binning et al., 2014). The ability to exploit such microhabitats for energy conservation enables fishes to inhabit energetically expensive areas – rich in food resources and optimal for breeding – without the high costs of increased locomotor effort. Hydrodynamically, the structural complexity of coral reefs disrupts water flow, creating areas of low energy behind the physical structures (Davis et al., 2021). These areas serve as not only flow refugia but also as strategic points for feeding. The slowed water carries plankton and other nutrients at reduced speeds, facilitating easier capture by the fish. Moreover, the reef's complexity offers multiple ambush points for predatory fishes, reducing the energy they need to invest in pursuing prey (Fulton and Bellwood, 2011).

Forage fishes, such as sardines, anchovies and herring, exhibit a collective behavior known as schooling, where groups of individuals swim in highly coordinated, synchronized formations (Weihs, 1973). This phenomenon is not merely a strategy for predator evasion or enhanced foraging efficiency but also a critical adaptation for energy conservation (Berio et al., 2023; Svendsen et al., 2003; Herskin and Steffensen, 1998). The dynamics of schooling involve significant energetic benefits to the fish within the school, often due to complex interactions that are influenced by the hydrodynamics of water (Li et al., 2019; Killen et al., 2012), as well as social interactions that are not dependent on hydrodynamics (Marras et al., 2015). Schooling enables forage fishes to exploit the vortices and wake generated by the leading fish, allowing trailing individuals to maintain the group swimming speed at a lower fin beat frequency (Svendsen et al., 2003) (Fig. 2F). This drafting, or slipstreaming, reduces the energy each fish expends to maintain speed and maneuver through the water (Weihs, 1973; Ashraf et al., 2017). When fish swim in a staggered or diamond formation, they may align themselves optimally to take advantage of these hydrodynamic effects across speeds (Weihs, 1973). Such positioning reduces the drag experienced by each fish, decreasing their oxygen consumption and overall energy expenditure (Zhang and Lauder, 2024; Di Santo, 2022). Studies have shown that fish in a school can reduce their energy use by up to 65% compared with solitary swimmers (many studies report a 10–20% advantage), thanks to reduced friction and the ability to ride in the forward-moving water created by their conspecifics (Herskin and Steffensen, 1998; Svendsen et al., 2003; Thandiackal and Lauder, 2023; Di Santo, 2024; Zhang and Lauder, 2024). The behavior showcases the remarkable ability of forage fishes to harness the physical properties of their aquatic environment to their advantage, in an energy-saving strategy.

## Implications for conservation

**Fig. 3.**
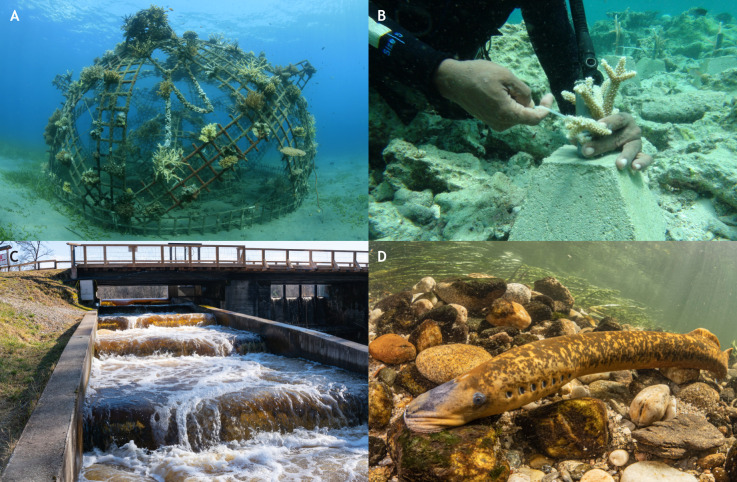
**Increasing habitat heterogeneity and complexity.** As fishes exploit 3D structures and friction with the substrate to reduce locomotor costs, efforts could be directed toward restoring habitat complexity as a key strategy for species conservation. Here, we provide a few examples: (A) installation of artificial structures where reefs have been destroyed, (B) coral farming and out-planting in the wild, (C) creation of passageways for fishes where habitats have been fragmented by structures, and (D) increase in substratum complexity to allow fishes to save energy during upstream migrations.

The exploitation of hydrodynamics and environmental structures by fishes to reduce locomotor costs highlights the need for rethinking targeted conservation efforts to prioritize restoration of habitat complexity as a key strategy for species conservation (Fig. 3). Although we do not suggest there is a universal definition of habitat complexity, we speculate that human action often tends to simplify ecosystems and alter their natural processes, and by doing so, perhaps decrease opportunities for species to use the physical habitat to their advantage. This section elaborates on strategic approaches to conservation that leverage our understanding of fish behaviors, focusing on coral reef restoration, enhancement of river processes and connectivity, and invasive species control as specific examples.

### Restoration of coral reef structures

The 3D structure and complexity of coral reefs have seen a dramatic decline in the past decades owing to bleaching and disease (Roff et al., 2020; Alvarez-Filip et al., 2009). These complex structures are essential not only for predator avoidance but also for enabling fishes to minimize energy expenditure while foraging and maintaining their position against currents. Restoration efforts must prioritize the re-establishment of this multi-dimensional complexity (Fig. 3A). Conservation strategies could support the protection and propagation of resilient coral species that contribute to structural diversity while being more likely to withstand warming waters (Graham et al., 2008) (Fig. 3B). Additionally, techniques such as deploying artificial reefs designed to mimic natural coral structures can provide immediate relief for many species (Jones et al., 2017; Pérez-Pagán and Mercado-Molina, 2018). Sunk wrecks and reef balls, artificial bio-engineered structures, can provide physical substratum for the re-establishment of corals and plants and re-create hydrodynamics conditions that fish can actively exploit to save energy. These structures may also provide larger benefits by helping to restore damaged mangroves or reducing the impacts of wave action on coastal environments.

### Enhancing river habitat connectivity for migratory species

Taxa such as salmonids, alosines and lampreys rely on various behavioral strategies to conserve energy during their migrations in freshwater, taking advantage of riverbed features, the natural hydraulic complexity, and even interactions with conspecifics. Understanding these behaviors is crucial for the management and conservation of these species, especially as they face pressure from human activities and climate change, which can alter river structures and flow regimes (Faucher et al., 2010). Barriers such as tidegates, dams and culverts severely disrupt fluvial ecosystems, altering flow regimes and blocking the migratory paths of many aquatic species. To mitigate these impacts, efforts must be made to enhance connectivity between aquatic habitats (Schlosser, 1991). This can include the removal of obsolete dams, the installation of effective fish passage solutions (Fig. 3C) adapted to specific behaviors such as drafting, attaching to the substrate, or even in-line swimming (Saadat et al., 2021; Thandiackal and Lauder, 2023), and also the restoration of natural fluvial processes that enhance riverbed heterogeneity and complex hydrodynamics (Bunn and Arthington, 2002; Poff and Zimmerman, 2010). In a natural river, fish will navigate the environmental complexity, likely responding to hydraulic parameters such as the total kinetic energy, the mean velocity of the flow, or the periodicity and scale of vortices shed by natural elements of the riverbed (Silva et al., 2018). A deeper understanding of the relationships between these parameters, the fish's preferences, and their energy-saving strategies can inform the engineering of fish passage structures and the restoration of altered river ecosystems. By aligning these structures with natural flow attributes, we can create optimal environments for effective and timely fish migrations (Lacey et al., 2012).

### Invasive species control: the case of the sea lamprey

As the attachment behavior allows lampreys to migrate upriver more efficiently, we suggest that promoting the ‘burst and attach’ behavior of lampreys in fishways, road-stream crossings and other artificial barriers characterized by fast flows may be beneficial to the conservation of lampreys in their native geographic range (Moser et al., 2021) (Fig. 3D). However, the introduction of invasive species such as the sea lamprey in the North American Great Lakes has had devastating effects on native fish populations (Docker et al., 2021). Effective control strategies for the sea lamprey have included targeted pesticide applications, trapping, and physical barriers preventing adults from accessing their river spawning grounds (Mills et al., 2003). However, sea lamprey barriers also fragment habitats for native species (McLaughlin et al., 2003; Dodd et al., 2003). Decreasing the ability of sea lamprey to attach to the substrate, and therefore making them swim harder to move upstream, may prove an effective strategy for reducing their dispersal rates in tributaries of the Great Lakes, as well as their reproductive success. Efforts to improve the design of lamprey barriers are currently underway, for example by combining high flow velocities and a substrate that inhibits attachment, in the hope of achieving selective passage, i.e. excluding lampreys while allowing passage for native fishes (Zielinski et al., 2019; Rahel, 2013).

## Conclusions

In this Commentary, we elaborate on the adaptive behaviors fishes exhibit to enhance locomotor efficiency at low and high speeds. Contrary to viewing fishes as mere ‘puppets of the environment’ (Krehl and Soetbeer, 1899), we present them as active exploiters of their habitats, employing strategies that circumvent traditional energetic constraints to conserve energy. By utilizing behaviors such as rolling, surfing, walking, hitchhiking and drafting, fishes demonstrate a sophisticated ability to optimize energy use, which is critical to their survival and evolutionary success. We stress the ecological significance of such behaviors, advocating for conservation strategies that prioritize the restoration of habitat complexity to support these locomotor behaviors.
